# Significantly enhanced thermal conductivity of indium arsenide nanowires via sulfur passivation

**DOI:** 10.1038/s41598-017-13792-4

**Published:** 2017-10-16

**Authors:** Yucheng Xiong, Hao Tang, Xiaomeng Wang, Yang Zhao, Qiang Fu, Juekuan Yang, Dongyan Xu

**Affiliations:** 1 Department of Mechanical and Automation Engineering, The Chinese University of Hong Kong, Shatin, New Territories, Hong Kong Special Administrative Region, China; 2 School of Mechanical Engineering and Jiangsu Key Laboratory for Design and Manufacture of Micro-Nano Biomedical Instruments, Southeast University, Nanjing, 210096 China

## Abstract

In this work, we experimentally investigated the effect of sulfur passivation on thermal transport in indium arsenide (InAs) nanowires. Our measurement results show that thermal conductivity can be enhanced by a ratio up to 159% by sulfur passivation. Current-voltage (I-V) measurements were performed on both unpassivated and S-passivated InAs nanowires to understand the mechanism of thermal conductivity enhancement. We observed a remarkable improvement in electrical conductivity upon sulfur passivation and a significant contribution of electrons to thermal conductivity, which account for the enhanced thermal conductivity of the S-passivated InAs nanowires.

## Introduction

With a narrow band gap and high electron mobility, InAs nanowires have attracted tremendous attention in recent decades as promising materials for gas sensors, high-speed transistors, and infrared photodetectors^1–5^. Due to the existence of a large density of surface states that originate from the unstable native oxide, the surface Fermi level is pinned above the conduction band, resulting in a surface accumulation layer of electrons^6^. Meanwhile, the surface electron mobility will get degraded by electron scattering induced by this surface charge layer^7–9^. Such effect is particularly severe in InAs nanowires due to the large surface-to-volume ratio. Over the past decades, surface passivation has been commonly adopted to passivate the InAs surface with a sulfur-containing monolayer, leading to enhanced surface electron mobility or concentration^8–13^. It has been reported that surface passivation with 1-octadecanethiol (ODT) or aromatic thiolate (ArS^-^)-based molecular monolayer can effectively enhance the electron mobility in InAs-nanowire-based field effect transistors^8,9^. On the other hand, sulfur passivation with either sulfur compounds or molecular sulfur has been shown to preserve the surface charge accumulation layer, leading to the increase of surface electron concentration^10–13^. Numerous works have been conducted on the effect of sulfur passivation on electrical^8–13^ and optical properties^14^ of InAs thin films or nanowires, but its effect on thermal transport properties has yet to be studied. Since electrons may contribute to a significant portion of thermal conductivity, it is fundamentally interesting to investigate how sulfur passivation affects thermal transport in InAs nanowires. On the other hand, from the point of view of thermal management, thermal conductivity of InAs nanowires is a crucial factor in determining the life-time and reliability of InAs-nanowire-based devices. A high thermal conductivity is desirable for better heat dissipation of InAs-based electronic devices.

In this study, we experimentally investigated the effect of sulfur passivation on thermal transport in InAs nanowires by comparing thermal conductivity values of the same nanowire with and without sulfur passivation. In the experiment, a uniform InAs nanowire with a length of tens of microns was cut into two segments. One segment was transferred onto a suspended microdevice for thermal conductivity measurement (unpassivated). The other segment was first sulfur-passivated as described in the methods section and then also transferred onto a microdevice for thermal measurement (S-passivated). The detailed sample preparation procedures are given in Fig. S1 (Supplementary Information). It was shown that thermal conductivity could be enhanced by a ratio up to 159% by sulfur passivation. I-V measurements were performed on both unpassivated and S-passivated InAs nanowires by using four-probe microdevices to understand the mechanism of thermal conductivity enhancement. We observed a remarkable improvement in electrical conductivity upon sulfur passivation and a significant contribution of electrons to thermal conductivity, which account for the enhanced thermal conductivity of the S-passivated InAs nanowires. Our study provides a facile approach to enhance thermal conductivity of InAs nanowires, which could facilitate heat removal in practical applications.

## Results and Discussion

### As-synthesized InAs nanowires

**Figure 1:**
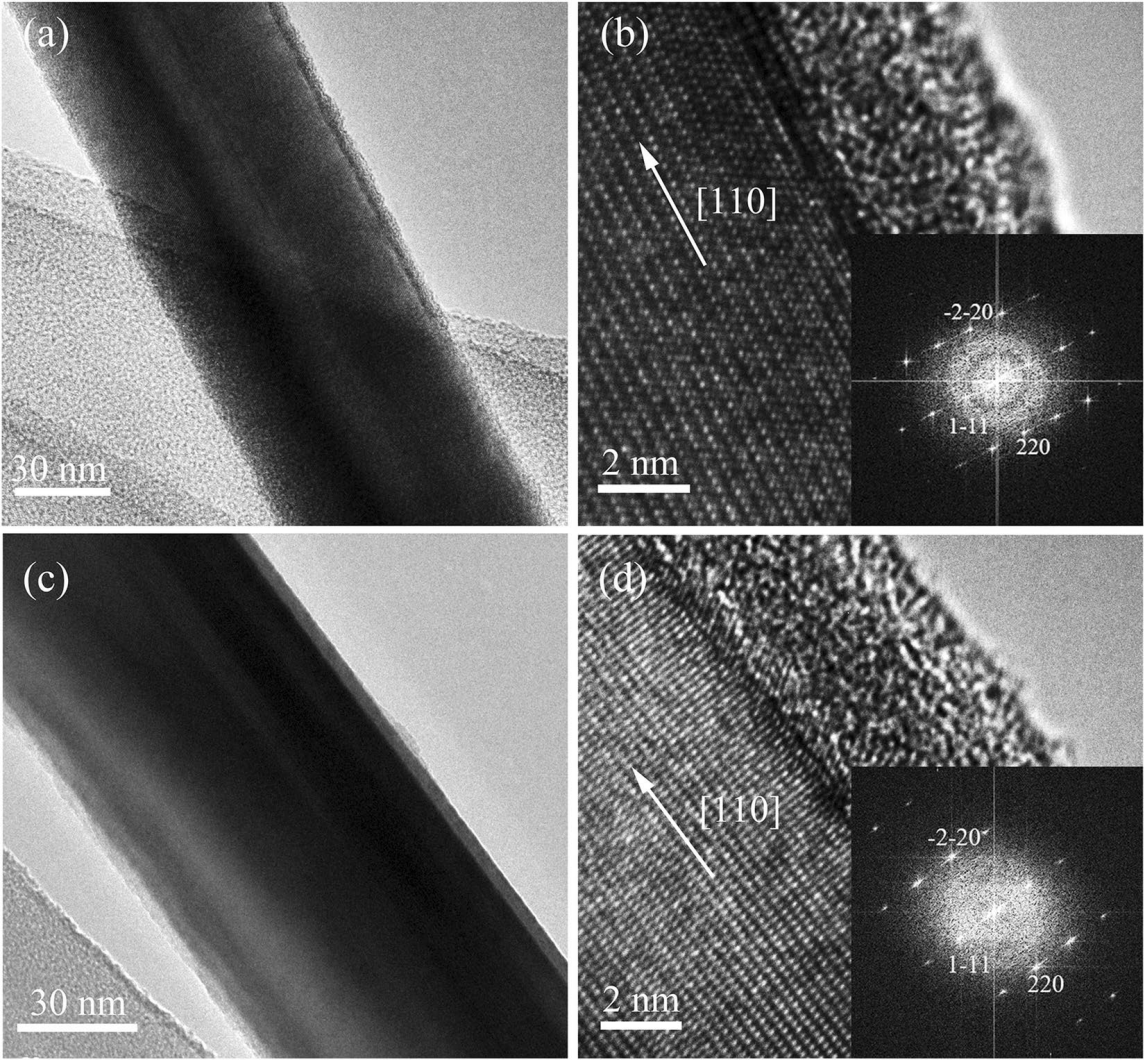
Structural characterization of the unpassivated and S-passivated segments of an InAs nanowire (S1). (**a**,**b**) TEM and HRTEM images of the unpassivated segment of S1; (**c**,**d**) TEM and HRTEM images of the S-passivated segment of S1. The insets of (**b** and **d**) show the selected area electron diffraction patterns. The growth direction is [110].

The as-synthesized InAs nanowires have a diameter ranging between 40 and 200 nm and a length of tens of microns. Figure 1 shows the transmission electron microscopy (TEM) and high-resolution TEM (HRTEM) images of the unpassivated and S-passivated segments of a typical InAs nanowire (sample S1). The synthesized InAs nanowire has a zinc blende crystal structure and grows in the [110] direction. The TEM study shows that the wire has a uniform diameter along the growth direction. A rather smooth amorphous layer with a thickness of a few nanometers was observed on the surface of both unpassivated and S-passivated segments.

**Figure 2:**
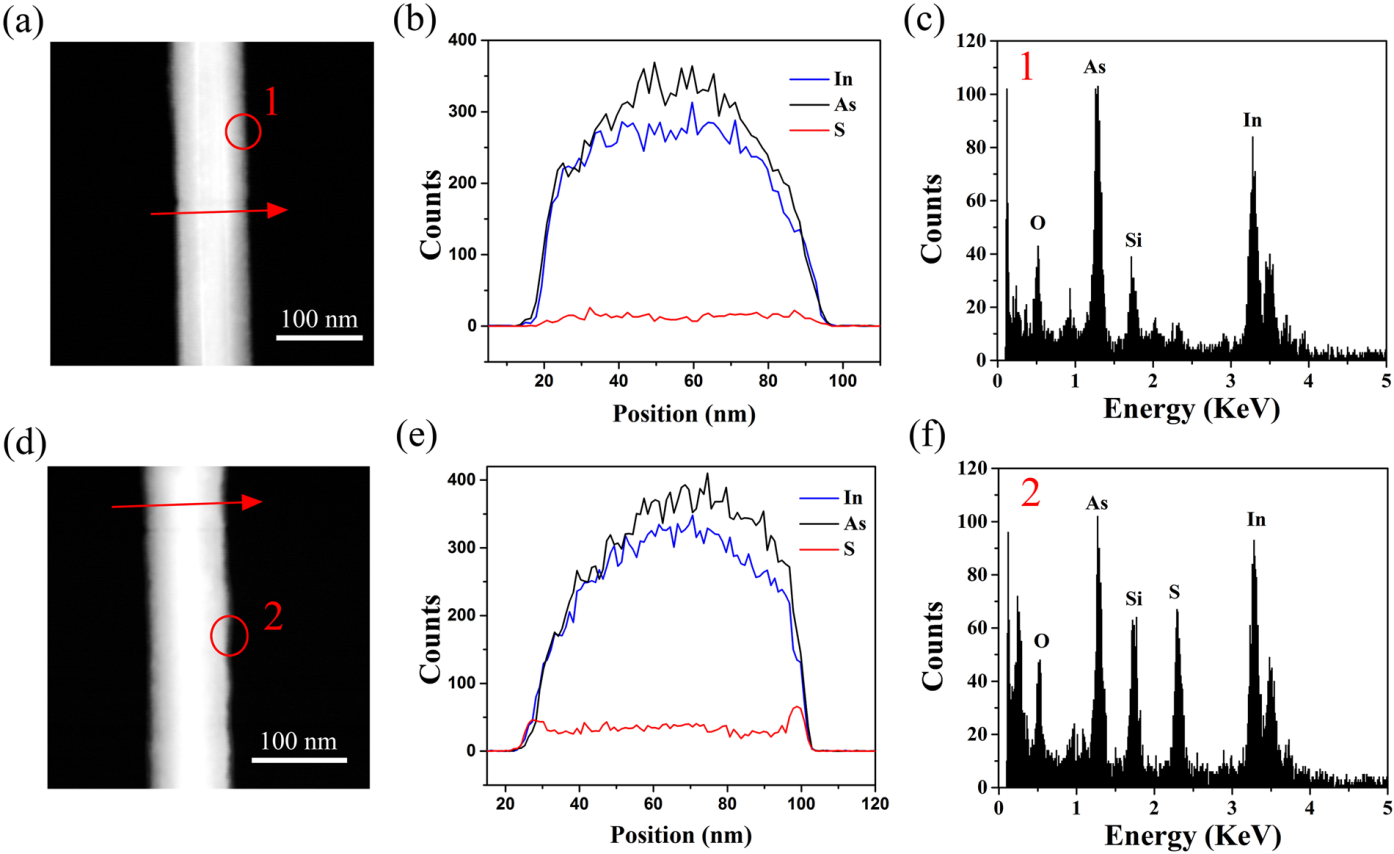
Chemical composition analyses of the unpassivated and S-passivated segments of S1. (**a**–**c**) The high-angle annular dark field (HAADF) image, elemental distribution, and HRTEM/EDS results for the unpassivated segment of S1; (**d**–**f**) HAADF image, elemental distribution, and HRTEM/EDS results for the S-passivated segment of S1. The red arrows in (**a** and **d**) show the line profile where STEM/EDS was taken; and the red circles on the nanowire surface indicate where HRTEM/EDS was taken.

The chemical composition of InAs nanowires was examined by using scanning transmission electron microscopy/energy dispersive X-ray spectrometry (STEM/EDS) and HRTEM/EDS. HRTEM/EDS analysis at the center of S1 (Fig. S2, Supplementary Information) indicates that the In:As ratio is close to 1:1. Line-profile analysis using STEM/EDS clearly shows that the S intensity of the unpassivated segment (Fig. 2b) is negligible and substantially higher S intensity is observed for the S-passivated segment (Fig. 2e). In Fig. 2e, the S intensity at the lateral surface is higher than that at the center, suggesting the formation of a sulfur layer on the nanowire surface. HRTEM/EDS analysis was also performed on the nanowire surface, as indicated by red circles in Fig. 2a and d. The clear S peak in the HRTEM/EDS result of the S-passivated segment also confirms the successful passivation of the nanowire surface with S atoms (Fig. 2f). On the other hand, it has been reported that (NH_4_)_2_S solution can effectively remove native oxides from the InAs surface during the sulfur passivation process^15–17^. However, in our study, an O peak was still observed in the HRTEM/EDS spectrum of the S-passivated segment (Fig. 2f). Moreover, as mentioned above, HRTEM image revealed an amorphous layer with a thickness of a few nanometers on the surface of the S-passivated segment. We speculate that the O signal on the surface of the S-passivated segment might be due to the incomplete sulfur passivation or re-oxidation of the surface after exposure in air for a long time. In addition, since the sulfur passivation was performed in the aqueous (NH_4_)_2_S solution, NH_4_OH might be formed on the nanowire surface, which could also contribute to the O signal in the EDS spectrum.

**Figure 3:**
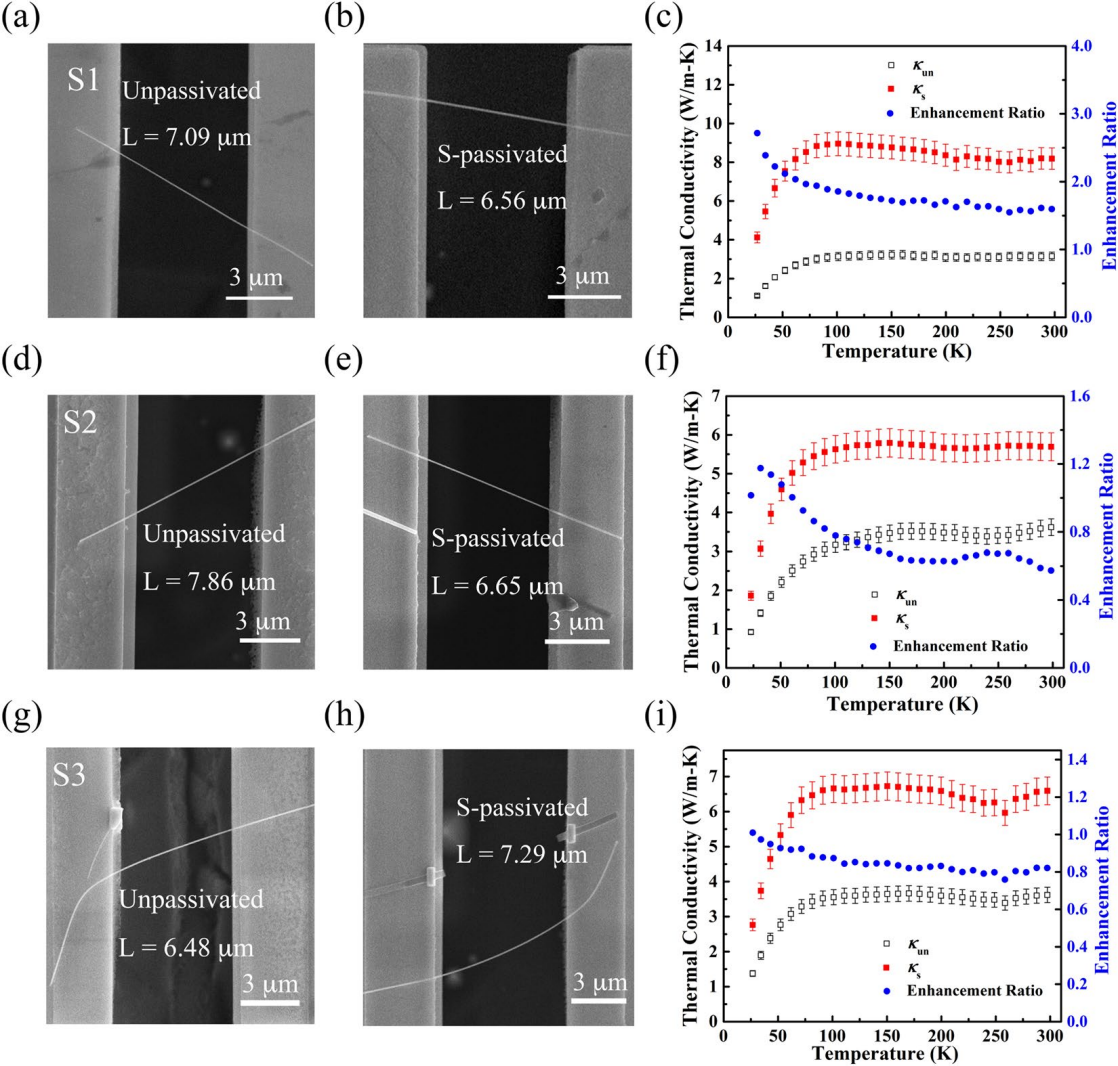
Scanning electron microscopy (SEM) images and the measured thermal conductivity of three sets of unpassivated and S-passivated InAs nanowires. The enhancement ratio of thermal conductivity, defined as (*κ*_s_ − *κ*_un_)/*κ*_un_, is shown on the right axis of (**c, f** and **i**). (**a**,**b**,**c**) for S1; (**d**,**e**,**f**) for S2; and (**g**,**h**,**i**) for S3. The diameters of S1, S2, and S3 are 76 nm, 86 nm, and 88 nm, respectively.

Thermal conductivity of three sets of InAs nanowires (unpassivated and S-passivated segments) was measured in the temperature range from 20 to 300 K (Fig. 3). At room temperature, thermal conductivity of the unpassivated segment is approximately one seventh of the corresponding value of the bulk InAs^18^, which is consistent with the previously reported results^19^. Interestingly, all three sets of InAs nanowires reveal a remarkable enhancement in thermal conductivity after sulfur passivation. The enhancement ratio of thermal conductivity is defined as (*κ*_s_ − *κ*_un_)/*κ*_un_, where *κ*_s_ and *κ*_un_ are thermal conductivity of the S-passivated segment and the counterpart of the unpassivated segment. As seen in Fig. 3, at room temperature, the enhancement ratios are 159%, 57%, and 82% for S1, S2, and S3, respectively. Our measurement results show a general trend of smaller enhancement ratio for nanowires with larger dimeter as seen in Fig. S3b (Supplementary Information). It is worth noting that the over 50% enhancement is well beyond the measurement uncertainty (Supplementary Information). On the other hand, as seen in TEM images in Fig. 1, the diameters of two segments of S1 are very close to each other (78 nm for the unpassivated segment and 74 nm for the S-passivated one). It is clear that thermal conductivity enhancement cannot be explained by the diameter variation.

When comparing thermal conductivity of the unpassivated and S-passivated segments, the effect of contact thermal resistance (*R*_c_) needs to be taken into account carefully, which can lead to underestimated thermal conductivity for the measured nanowires. In our previous studies^20,21^, we have demonstrated that *R*_c_ can be extracted by fitting the total thermal resistance data (*R*_tot_) of the same nanowire at different suspended lengths (*L*_s_). In this study, we adopted the same approach and the results are presented in Fig. S4 (Supplementary Information). The extracted *R*_c_ contributes ~25% of *R*_tot_ at room temperature. At an extreme case, even if we assume that the passivation process can reduce *R*_c_ to zero, thermal conductivity can only be enhanced by ~33%. Therefore, the reduction in contact thermal resistance cannot account for the more than 50% enhancement ratio of thermal conductivity for the S-passivated InAs nanowires. On the other hand, no obvious difference in surface roughness was observed for the unpassivated and S-passivated segments of S1 (Fig. 1). Thus, we believe that the markedly enhanced thermal conductivity is unlikely due to the change of surface roughness by sulfur passivation. We also conducted a control experiment with only deionized (DI) water and no obvious change in thermal conductivity is observed after the DI water treatment as shown in Fig. S5 (Supplementary Information).

It has been reported that sulfur passivation can effectively enhance electron transport properties of the InAs surface by improving either electron mobility^8,9^ or concentration^10–13^. It is natural to guess that the enhanced thermal conductivity of the S-passivated InAs nanowires results from the improved electrical properties by sulfur passivation. To verify this hypothesis, I-V measurements were performed on both unpassivated and S-passivated segments of S3 by using four-probe microdevices. Unfortunately, as shown in Fig. S6c (Supplementary Information), nonlinear IV curves were observed for both segments presumably due to the existence of the amorphous oxide layer on the nanowire surface. To quantitatively estimate the contribution of electrons to thermal conductivity of InAs nanowires, we further studied the InAs nanowire etched by the hydrofluoric solution (HF).

### HF-etched InAs nanowire

An InAs nanowire was first transferred onto a TEM grid. Then, the grid was dipped in 0.05 wt% HF solution for 30 s to remove the amorphous oxide layer. Right after the HF etching, the nanowire was cut into two segments. Sulfur passivation was performed on one segment. Next, both the unpassivated and S-passivated segments were transferred onto the four-probe microdevices. The contacts between the nanowire and Pt electrodes were treated with the electron beam induced deposition (EBID) of Pt/C composites to enhance both electrical and thermal contacts. After the EBID treatment, thermal and electrical measurements were conducted for both segments.

**Figure 4:**
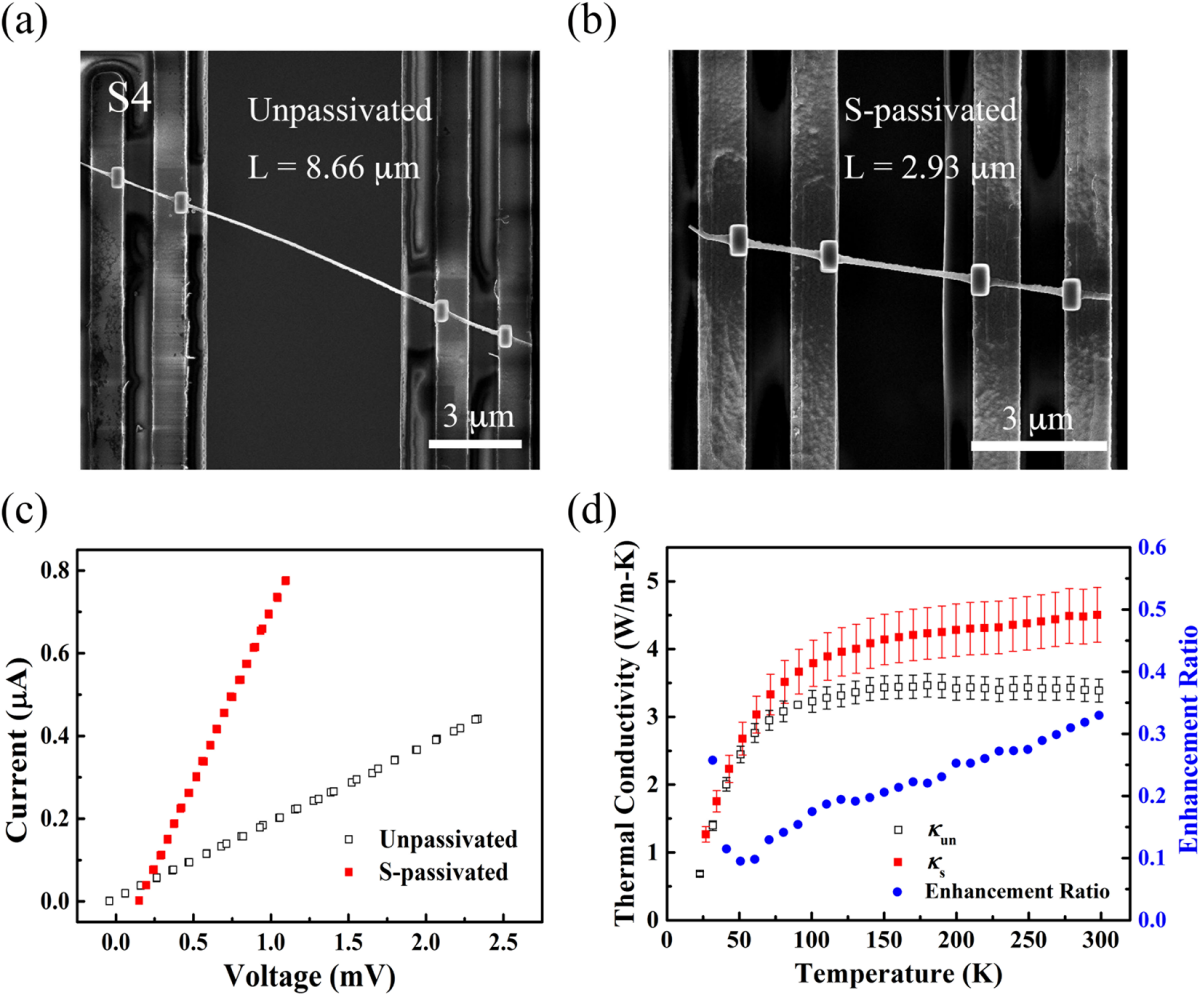
SEM images of S4 on the four-probe microdevices: (**a**) The unpassivated segment; (**b**) The S-passivated segment. (**c**) The measured I-V curves at 300 K. (**d**) Thermal conductivity of the unpassivated and S-passivated segments of S4. The enhancement ratio is shown on the right axis. The diameter of S4 is 98 nm.

The results for the HF-etched InAs nanowire (S4) are given in Fig. 4 and Table 1. Linear I-V curves were observed for both the unpassivated and S-passivated segments, suggesting ohmic contacts between the nanowire and Pt electrodes. At 300 K, thermal conductivity of the S-passivated segment is approximately 33% higher than that of the unpassivated segment. In general, thermal conductivity of a semiconductor comprises two parts: lattice thermal conductivity (${\kappa }_{{\rm{ph}}}$) and electronic thermal conductivity (${\kappa }_{{\rm{e}}}$). ${\kappa }_{{\rm{e}}}$ can be estimated by using the Wiedemann-Franz law:

1 $${\kappa }_{{\rm{e}}}=L\sigma T,$$

where *L* is the Lorenz number and commonly set as the Sommerfeld value of 2.44 × 10^−8^ WΩK^−2^, $\sigma$ is electrical conductivity, and *T* is the absolute temperature. As shown in Fig. S7a (Supplementary Information), the electrical conductivity of both the unpassivated and S-passivated segments of S4 shows a decreasing trend with temperature, exhibiting metallic behavior. Thus, it is valid to use the Wiedemann-Franz law and the Sommerfeld value to estimate ${\kappa }_{{\rm{e}}}$ for S4. As listed in Table 1, for the unpassivated segment, the electronic thermal conductivity is estimated to be 1.53 W/m-K, which contributes roughly 45% to the total thermal conductivity. After sulfur passivation, the electrical conductivity is enhanced by 50% and the estimated electronic thermal conductivity is 2.31 W/m-K, leading to a thermal conductivity enhancement ratio of ~23%, which is very close to the experimentally obtained value of 33%. It is worth noting that the lattice thermal conductivity, calculated by subtracting the electronic thermal conductivity from the total thermal conductivity, is 2.19 W/m-K at 300 K for the S-passivated segment, which is close to the counterpart of the unpassivated segment (1.86 W/m-K). This result seems reasonable and further justifies the applicability of the Wiedemann-Franz law in InAs nanowires. The significant contribution of electrons to thermal conductivity of InAs nanowires has been previously reported^22^ and is attributed to the presence of the surface charge accumulation layer. Recent studies have shown that sulfur passivation preserves the surface charge accumulation layer and effectively enhance surface electron concentration or mobility^8–13^. As such, we believe that the enhanced thermal conductivity of the S-passivated InAs nanowires can be attributed to the significant contribution of electrons to thermal conductivity and the enhancement of electron transport properties by sulfur passivation. In addition, we expect that electron-phonon scattering and interfacial scattering between crystalline InAs core and thin amorphous surface layer will also affect both electron and phonon transport in InAs nanowires. However, our thermal and electrical characterization could not differentiate their roles in the enhanced thermal conductivity of the S-passivated InAs nanowires.

**Table 1:** The total thermal conductivity (${\kappa }_{{\rm{tot}}}$), electrical conductivity ($\sigma$), electronic thermal conductivity (${\kappa }_{{\rm{e}}}$), lattice thermal conductivity (${\kappa }_{{\rm{ph}}}$), and ${\kappa }_{{\rm{e}}}/{\kappa }_{{\rm{tot}}}$ for the unpassivated and S-passivated segments of S4 at 300 K.

		${{\boldsymbol{\kappa }}}_{{\bf{tot}}}$ (W/m-K)	${\boldsymbol{\sigma }}$ (10^5^ S/m)	${{\boldsymbol{\kappa }}}_{{\bf{e}}}$ (W/m-K)	${{\boldsymbol{\kappa }}}_{{\bf{ph}}}$ (W/m-K)	${{\boldsymbol{\kappa }}}_{{\bf{e}}}/{{\boldsymbol{\kappa }}}_{{\bf{tot}}}$
S4	Unpassivated	3.39	2.11	1.53	1.86	45%
	S-passivated	4.50	3.19	2.31	2.19	51%

**Figure 5:**
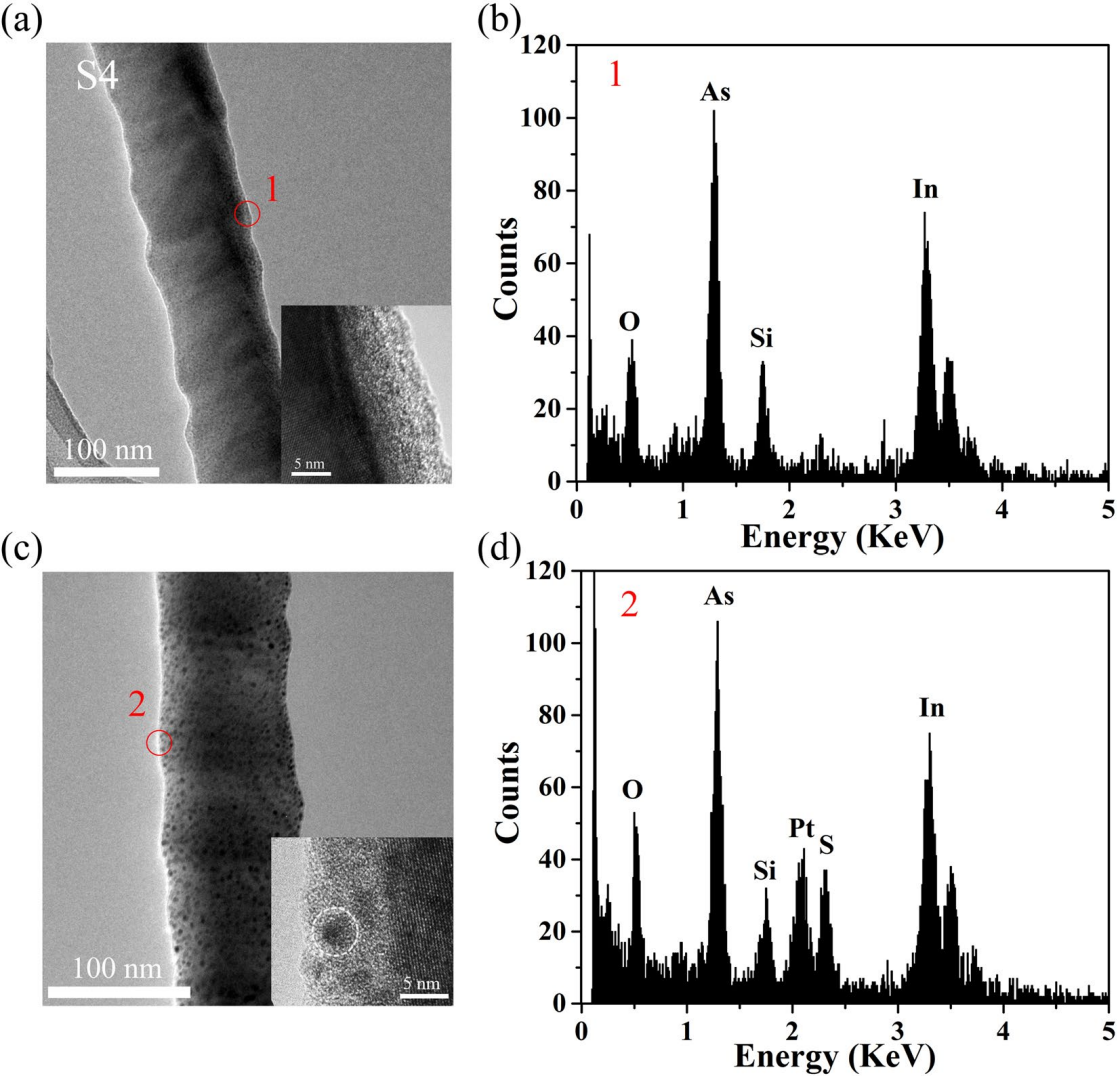
TEM images and HRTEM/EDS results of S4: (**a**,**b**) The unpassivated segment; (**c**,**d**) The S-passivated segment. The insets in (**a** and **c**) show the HRTEM image. The red circles on the nanowire surface indicate the location where EDS was taken. The white dashed circle in the inset of (**c**) refers to the Pt particle.

The HRTEM/EDS results of S4 are presented in Fig. 5. A Pt peak is observed in the EDS spectrum of the S-passivated segment, which might come from the Pt/C deposition. On the other hand, isolated black dots are observed on the nanowire surface (Fig. 5c), which are likely Pt nanoparticles. Metal decoration has been proven to be an effective approach to tune electrical properties of InAs nanowires^3,23^. Gold (Au) decoration was reported to act as an electron withdrawer in InAs-nanowire-based field effect transistors, resulting in the reduction of both electron mobility and electron concentration^23^. The work function of Pt (5.12–5.93 eV) is close to that of Au (5.31–5.47 eV), both of which are higher than the Fermi level of the intrinsic n-type InAs (5.0 eV). Therefore, we hypothesize that, similar to the effect of Au decoration, Pt nanoparticles on the nanowire surface might also reduce the electron concentration and mobility, which contradicts the effect of sulfur passivation on the thermal conductivity of InAs nanowires.

## Conclusion

In summary, we experimentally demonstrated that sulfur passivation could enhance thermal conductivity of InAs nanowires by a ratio up to 159% at room temperature. The enhanced thermal conductivity is attributed to the significant contribution of electrons to thermal conductivity and the enhancement in electron transport properties of InAs nanowires by sulfur passivation. Our study provides an effective approach to enhance thermal transport in InAs nanowires, which has important implications for thermal management of InAs-nanowire-based electronic devices.

## Methods

### Synthesis of InAs nanowires

High quality single crystalline InAs nanowires were synthesized via a vapor-liquid-solid method in a three-zone tube furnace. InAs powders (99.999%, Sigma-Aldrich) were placed in the hot center of the tube furnace. A silicon (Si) substrate coated with Au nanoparticles with a diameter of ~10 nm was placed downstream in the same quartz tube. The tube was initially evacuated and then Argon gas was passed through the tube as the carrier gas. The pressure was maintained at approximately 10–20 mbar during the synthesis process. The precursor was heated up to 630 °C at a ramping rate of approximately 40 °C/min and was kept at this temperature for 30 min. InAs nanowires were then obtained on the Si substrate.

### Sulfur passivation

Analytical reagent grade ammonium sulfide solution [(NH_4_)_2_S, J&K Scientific Ltd.] was used to prepare the sulfur passivation solution. Following a recipe in the literature^24^, the solution was first diluted from 20% to 10% with the deionized water. Then, the sample was dipped in 10% (NH_4_)_2_S solution for 20 min at room temperature. The S-passivated segment was blown dry and transferred onto the microdevice.

### Thermal and electrical characterization

Thermal conductivity of InAs nanowires was characterized through a suspended thermal bridge method^25^ that has been widely used to determine thermal transport properties of one-dimensional nanostructures. As illustrated in Fig. S8 (Supplementary Information), the microdevice consists of two suspended membranes separated by several microns. An individual nanowire was aligned between two membranes by using a tungsten needle. Each membrane serves as heat source or heat sink in thermal conductivity characterization. Thermal conductance of the nanowire was determined by solving the heat transfer model of the entire system. With the diameter and the suspended length of the nanowire extracted from the SEM images, thermal conductivity of the nanowire can be extracted. In this study, two-probe microdevices were used for thermal measurements. The Pt electrode on each membrane has a width of 3 μm, which is beneficial for thermal measurements owing to the large contact area between the nanowire and the electrode.

A four-probe microdevice with two Pt electrodes on each membrane, as illustrated in Fig. S8b (Supplementary Information), was used to characterize both electrical conductivity and thermal conductivity of InAs nanowires. The contacts between the nanowire and Pt electrodes were treated with EBID of Pt/C composites to enhance both electrical and thermal contacts. In electrical measurements, a DC current was passed through two outer electrodes, which was recorded using a low-noise current preamplifier (SR570, Stanford Research Systems). The voltage drop between two inner electrodes was measured using a low-noise voltage preamplifier (SR560, Stanford Research Systems). The electrical conductivity of the InAs nanowire was extracted from the measured electrical resistance, the diameter of the nanowire, and the suspended length between two inner electrodes.

## Electronic supplementary material

Supplementary Information
