# Supplementary material for: Significantly enhanced thermal conductivity of indium arsenide nanowires via sulfur passivation
Source: Sci Rep. 2017 Oct 16;7:13252. doi: 10.1038/s41598-017-13792-4 (PMC5643329; doi:10.1038/s41598-017-13792-4)
Supplement: Supplementary file 1 — Supplementary Information [file 41598_2017_13792_MOESM1_ESM.pdf]

Supplementary Information

Significantly enhanced thermal conductivity of indium arsenide nanowires via sulfur passivation

Yucheng Xiong[1], Hao Tang[1], Xiaomeng Wang[1], Yang Zhao[2], Qiang Fu[1], Juekuan Yang[2] & Dongyan Xu[1]

[1]Department of Mechanical and Automation Engineering, The Chinese University of Hong Kong, Shatin, New Territories, Hong Kong Special Administrative Region, China.

[2]School of Mechanical Engineering and Jiangsu Key Laboratory for Design and Manufacture of Micro-Nano Biomedical Instruments, Southeast University, Nanjing 210096, China.

Correspondence and requests for materials should be addressed to D.X. (e-mail: dyxu@mae.cuhk.edu.hk)

I. Sample Preparation and Experimental Procedures

Under an optical microscope, a uniform InAs nanowire with a length of tens of microns was first cut into two segments by using a sharp needle mounted on a micromanipulator. One segment was directly transferred onto a microdevice, as shown in Fig. S1b. For sulfur passivation, a drop of 10% $(NH_4)_2S$ solution was dripped on a poly-(dimethylsiloxane) (PDMS) substrate. The other segment was picked up with the needle and dipped in the solution drop for 20 min, as shown in Fig. S1c. Next, the S-passivated segment was blown dry and aligned onto a microdevice. After the thermal measurement, the scanning electron microscopy (SEM) image of the nanowire was taken to extract the suspended length and the diameter of the nanowire. Then, both the unpassivated and S-passivated segments were transferred onto a grid for the high-resolution transmission electron microscopy/energy dispersive X-ray spectrometry (HRTEM/EDS) study.

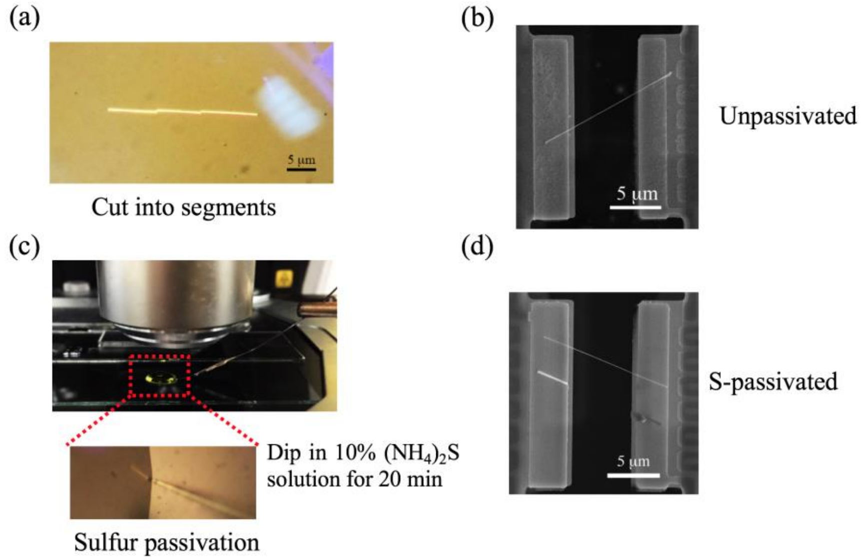

Figure S1. The schematic diagram of the sample preparation process.

II. EDS Results of S1

Figure S2 gives the EDS spectra and zoom detailing spectra of the unpassivated and S-passivated segments of S1. The EDS was taken at the center of the nanowire. The In:As ratio is close to 1:1. An S peak was observed in the EDS spectrum of the S-passivated segment (Fig. S2d), confirming the passivation of the surface with S atoms.

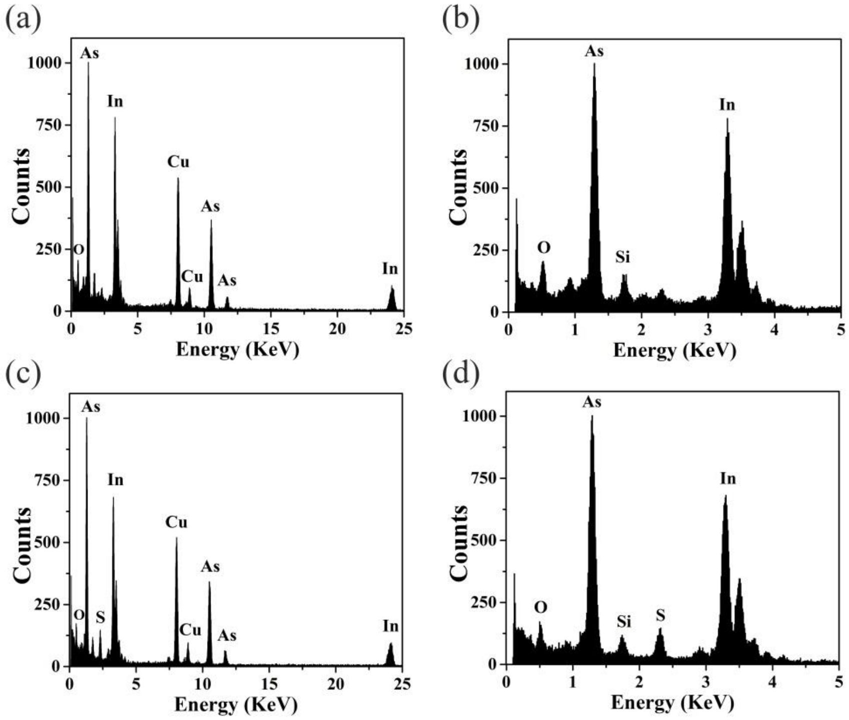

Figure S2. (a), **(b)** EDS and zoom detailing spectra of the unpassivated segment of S1; **(c)**, **(d)** EDS and zoom detailing spectra of the S-passivated segment of S1.

III. Additional Experimental Results and the Diameter Dependence of the Enhancement Ratio

Additional experimental results of an InAs nanowire with a diameter of 106 nm are given in Fig. S3a. Figure S3b summarizes the enhancement ratio for InAs nanowires with different diameters, which shows a general trend of smaller enhancement ratio for nanowires with larger dimeter. It is noticed that the enhancement ratio fluctuates a bit for nanowires with the diameter of 86 nm and 88 nm, which might be due to the measurement uncertainty and variation in electrical conductivity of different nanowires.

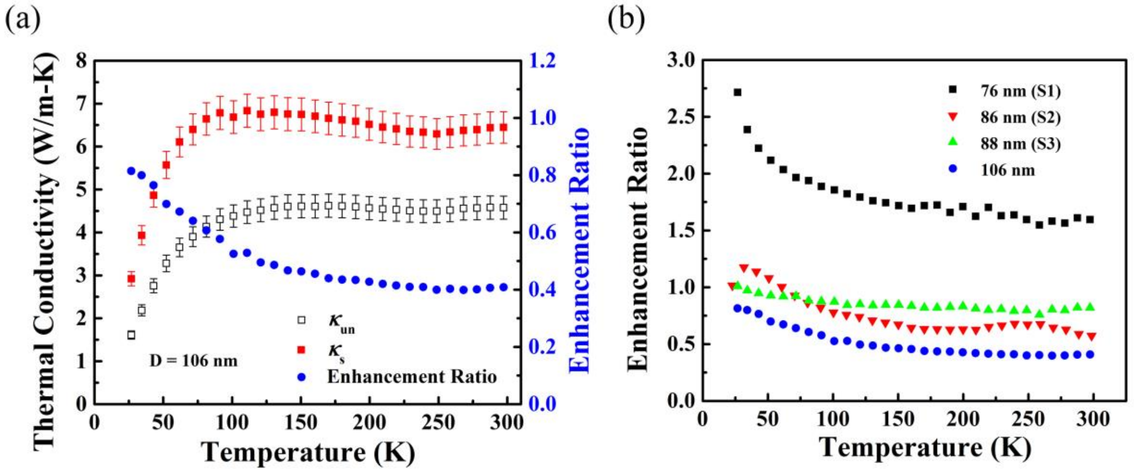

Figure S3. (a) Thermal conductivity of the unpassivated segment (κ_{un}) and the S-passivated segment (κ_s) of an InAs nanowire with a diameter of 106 nm. The enhancement ratio of thermal conductivity, defined as $(\kappa_s - \kappa_{un})/\kappa_{un}$, is shown on the right axis. **(b)** The enhancement ratio for InAs nanowires with different diameters.

IV. Uncertainty Analysis

The relative uncertainty of thermal conductance was evaluated using a Monte Carlo method,[1] which is less than 2% for the nanowires measured in this work. The suspended length and the diameter of the nanowires were measured from the SEM images, and the errors were estimated to be 0.25 μm and 2 nm, respectively. The relative uncertainty in thermal conductivity is less than 10% for all the measured samples, evaluated by the standard approach of the uncertainty propagation, which is much smaller than the thermal conductivity enhancement ratio induced by sulfur passivation.

V. Contact Thermal Resistance

According to the fin heat transfer model,[2] the contact thermal resistance (R_c) can be assumed to be same in different measurements if the contact length of the nanowire with each membrane is long enough. Under this assumption, an InAs nanowire was measured for three times with suspended lengths (L_S) of 3.44 μm, 4.42 μm, and 6.63 μm, respectively. At each temperature, the measured total thermal resistance increases linearly with L_s, as shown in the inset of Fig. S4d, which in turn verifies the assumption that R_c is same in different measurements. The y-axis intercept of the linear fitting curve represents R_c between the nanowire and two membranes; and the slope is the thermal resistance per unit length of the nanowire. Figure S4e gives the ratio of R_c to the total thermal resistance (R_{tot}) measured with L_s = 6.63 μm. At room temperature, R_c contributes approximately 25% to R_{tot}.

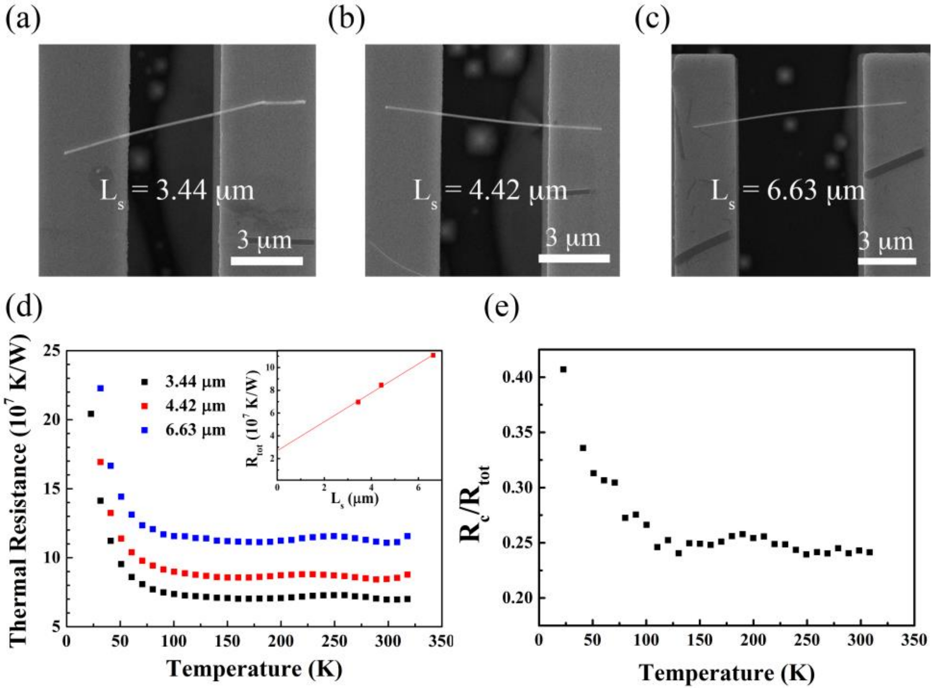

Figure S4. SEM images of an InAs nanowire with suspended lengths of **(a)** 3.44 μm, **(b)** 4.42 μm, and **(c)** 6.63 μm. **(d)** The measured total thermal resistance as a function of temperature for three different suspended lengths. The inset shows the total thermal resistance versus the suspended length at 300 K. **(e)** The ratio of R_c to R_{tot} measured with the suspended length of 6.63 μm.

VI. The Effect of the Deionized (DI) Water

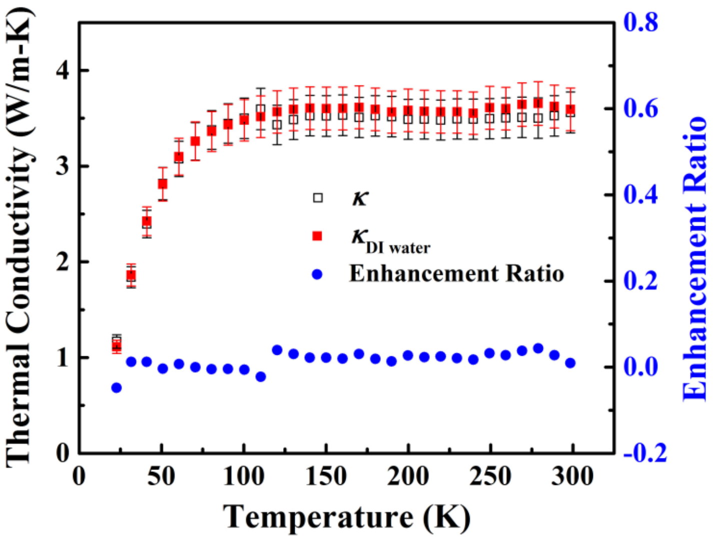

Figure S5. Thermal conductivity of an InAs nanowire with and without the DI water treatment. The enhancement ratio of thermal conductivity, defined as $(\kappa_{\mathrm{DI\ water}} - \kappa)/\kappa$, is shown on the right axis.

VII. Electrical Conductivity and Thermal Conductivity of S3 Measured on the Four-Probe Microdevices

To compare electrical conductivity of the unpassivated and S-passivated segments, the two segments of S3 were transferred onto the four-probe microdevices (Figs. S6a and S6b). Platinum/carbon (Pt/C) composites were deposited at contacts between the nanowire and Pt electrodes by the electron beam induced deposition (EBID) to enhance both electrical and thermal contacts. After that, I-V characteristics were measured as shown in Fig. S6c. The I-V curves of both unpassivated and S-passivated segments demonstrate a diode-like behavior presumably due to the existence of the amorphous oxide layer on the nanowire surface. Although it has been reported that ohmic contacts could be formed between the InAs nanowire and metal electrodes after sulfur passivation,[3,4] it was not observed for the S-passivated segment of S3 in our study, which might be caused by the incomplete sulfur passivation or re-oxidation of the surface after exposure in air for a long time. Importantly, the current of the S-passivated segment is over one order of magnitude higher than the counterpart of the unpassivated segment, indicating significantly enhanced electrical conductivity by sulfur passivation.

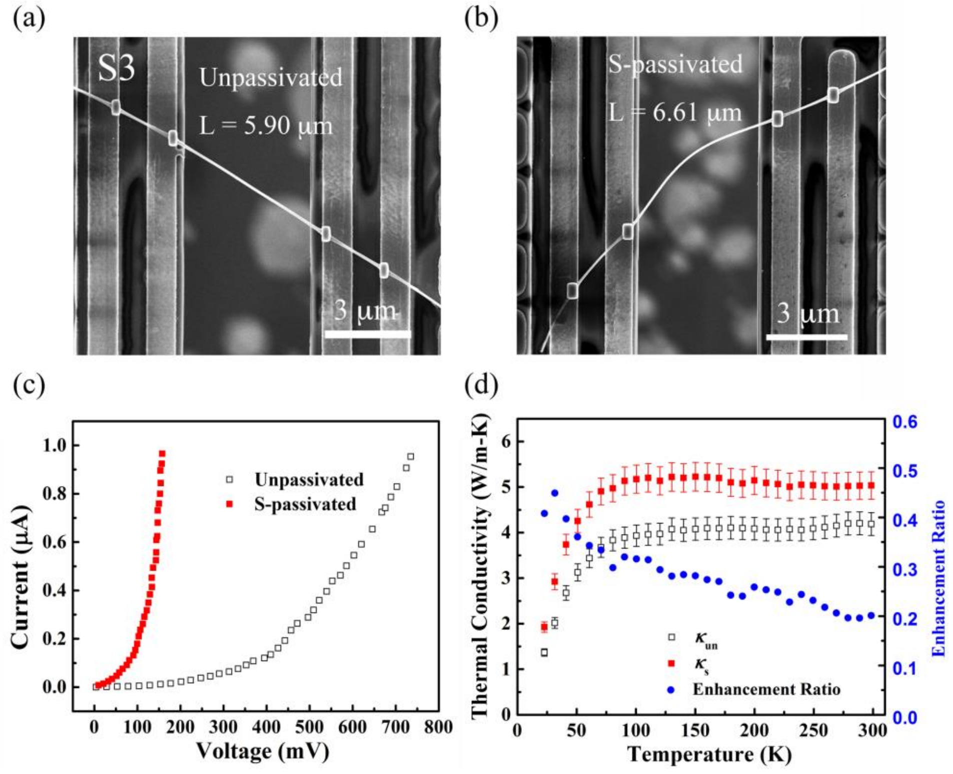

Figure S6. SEM images of S3 on the four-probe microdevices: **(a)** The unpassivated segment; **(b)** The S-passivated segment. **(c)** The measured I-V curves at 300 K. **(d)** Thermal conductivity of the unpassivated and S-passivated segments of S3 measured on the four-probe microdevices. The enhancement ratio is shown on the right axis.

We also measured thermal conductivity of two segments of S3 on the four-probe microdevices and results are shown in Fig. S6d. Thermal conductivity of the unpassivated segment of S3 obtained from the four-probe microdevice (Fig. S6d) is slightly higher than the value obtained from the two-probe microdevice (Fig. 3i), which is reasonable since Pt/C composites deposited

at contacts between the nanowire and Pt electrodes can effectively reduce the contact thermal resistance. However, thermal conductivity of the S-passivated segment of S3 obtained from the four-probe microdevice is much lower than that previously determined on the two-probe microdevice (Fig. 3i). As discussed in the main manuscript, we hypothesize that Pt contamination formed on the nanowire surface during the EBID process might reduce the electron concentration and mobility, which contradicts the effect of sulfur passivation on the thermal conductivity of InAs nanowires.

VIII. Electrical Conductivity and Thermal Conductivity of S4

Temperature-dependent electrical conductivity of S4 is plotted in Fig. S7a. For the S-passivated segment, we only have the electrical conductivity data in the temperature range of 250-300 K. The electrical conductivity of both the unpassivated and S-passivated segments of S4 shows a decreasing trend with temperature, exhibiting metallic behavior. Thus, it is valid to use the Wiedemann-Franz law and the Sommerfeld value to estimate κ_e for S4. Figure S7b shows the measured total thermal conductivity (κ_{tot_un} or κ_{tot_s}), the electronic thermal conductivity estimated from the Wiedemann-Franz law (κ_{e_un} or κ_{e_s}), and the lattice thermal conductivity (κ_{ph_un} or κ_{ph_s}) of S4. In the temperature range of 250 to 300 K, the extracted κ_{ph_un} and κ_{ph_s} are very close to each other, which is reasonable and to some extent justifies the use of the Wiedemann-Franz law.

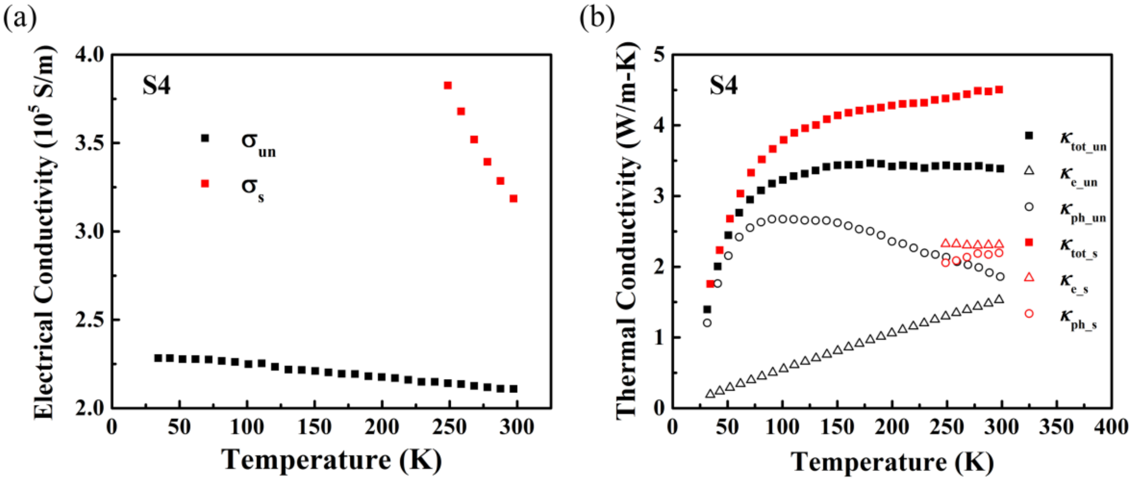

Figure S7. (a) Electrical conductivity of the unpassivated (σ_{un}) and S-passivated (σ_s) segments of S4; **(b)** Thermal conductivity of S4: κ_{tot_un}, κ_{e_un}, and κ_{ph_un} are the total, electronic, and lattice thermal conductivity of the unpassivated segment; κ_{tot_s}, κ_{e_s}, and κ_{ph_s} are the counterparts of the S-passivated segment.

IX. Microdevices for Thermal and Electrical Measurements

Figure S8 shows the SEM images of two-probe and four-probe microdevices with a nanowire bridging two suspended membranes. In this study, two-probe microdevices were used for thermal measurements. The platinum (Pt) electrode on each membrane has a width of 3 μm, which is beneficial for thermal measurements owing to the large contact area between the nanowire and the electrode. The four-probe microdevices were used for both electrical and thermal measurements.

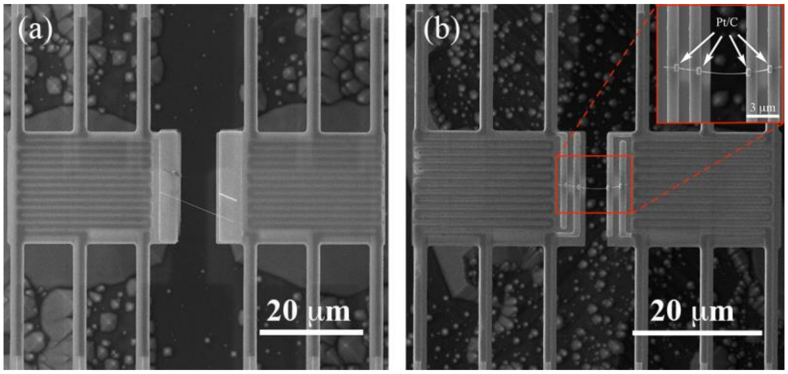

Figure S8. SEM images of microdevices with a nanowire bridging two suspended membranes: **(a)** Two-probe microdevice; **(b)** Four-probe microdevice.

References

1 Coleman H. W. & Steele W. G. Experimentation and uncertainty analysis for engineers. *John Wiley & Sons Inc.* (New York, 1999).

2 Yu, C. et al. Thermal contact resistance and thermal conductivity of a carbon nanofiber. *J. Heat Transfer* **128,** 234-239 (2006).

3 Suyatin, D. B., Thelander, C., Björk, M. T., Maximov, I. & Samuelson, L. Sulfur passivation for ohmic contact formation to InAs nanowires. *Nanotechnology* **18,** 105307 (2007).

4 Sourribes, M. J., Isakov, I., Panfilova, M. & Warburton, P. A. Minimization of the contact resistance between InAs nanowires and metallic contacts. *Nanotechnology* **24,** 045703 (2013).
